# Bacillary layer detachment with malignant choroidal tumors: a case series

**DOI:** 10.1186/s12886-023-02892-7

**Published:** 2023-04-06

**Authors:** Yousef Ahmed Fouad, Abdelrahman Gaber Salman, Doaa Maamoun Ashour, Mohamed Sabry Elkady, Noha Abdel-khalek, Mohamed Nowara, Weam Mohamed Ebeid

**Affiliations:** 1 Ophthalmology Department, Ain Shams University, Ramses st, Cairo, 11517 Cairo Egypt; 2 Clinical Oncology Department, Ain shams University, Cairo, Egypt; 3 Vitreoretinal Service, Al Mashreq Eye Center, Cairo, Egypt

**Keywords:** Bacillary Layer detachment, BALAD, Choroidal Melanoma, Choroidal Metastasis, Choroidal tumors

## Abstract

**Purpose:**

To study the incidence and characteristics of bacillary layer detachment (BALAD) occurring with the two most common choroidal malignancies, choroidal metastasis and choroidal melanoma.

**Methods:**

A retrospective multicentric record analysis. Eyes with a diagnosis of choroidal melanoma or choroidal metastasis that had good-quality fundus photography and spectral domain optical coherence tomography (OCT) scans of the macular and tumor regions allowing for delineation of the retinal layers were included for analysis. Qualitative image evaluation was done by two independent graders for the presence, location, and OCT features of BALAD, as well as any associated intraretinal or subretinal fluid. Demographic and clinical data were also retrieved.

**Results:**

Of the 11 eyes with choroidal metastasis and 7 eyes with choroidal melanoma that were included in the final analysis, 6 (54.5%) and 1 (14.3%) had BALAD, respectively. The BALAD co-localized with the subretinal fluid in all cases and with the intraretinal fluid in 1/3 cases (33.3%), was foveal in location in 3 eyes (42.9%), was overlying the tumor in 6 eyes (85.7%), and varied in number and size. Reflectivity within the BALAD was consistently higher than the vitreous and adjacent subretinal fluid, and discernable suspended hyperreflective particles were noted in 5 eyes (71.4%).

**Conclusion:**

BALAD is relatively common with choroidal metastasis. The OCT features described supplement our recognition of this new entity.

## Introduction

Bacillary layer detachment (BALAD) is a recently recognized optical coherence tomography (OCT) finding that describes a split within the inner segments of the photoreceptors, just posterior to the external limiting membrane (ELM) [1]. While not pathognomonic for a specific disease, BALAD has been increasingly associated with inflammatory and infiltrative choroidal pathologies and has been suggested to occur due to an influx of fluid into the subretinal space that exceeds the pump capacity of the retinal pigment epithelium (RPE) with an intact ELM [1–3].

Special attention has recently been given to studying BALAD as a prognostic indicator in exudative macular pathologies, especially neovascular age-related macular degeneration [2–5], and in inflammatory choroidal conditions such as Vogt-Koyanagi-Harada disease [6]. This may have led to an overrepresentation of these cases within the literature [1]. Isolated case reports have described BALAD with other ocular pathologies, where the incidence and spectrum of the finding is less clear, including blunt eye trauma [7, 8], the pachychoroid spectrum of disorders [9, 10], choroidal tuberculous granuloma [11], and other causes of choroiditis [12, 13]. Two case reports [10, 14] described the occurrence of BALAD with choroidal metastasis, and another one [15] described it with the rare entity of unilateral diffuse uveal melanocytic proliferation. Herein, we study the incidence and characteristics of BALAD occurring with the two most common choroidal malignancies: choroidal metastasis and choroidal melanoma.

## Methods

### Study design and setting

This observational multicentric retrospective analysis involved two centers, the ophthalmology department at Ain Shams university hospitals and a private eye center (Al Mashreq Eye Center), both located in Cairo, Egypt. The study was approved by the research ethics committee at the Faculty of Medicine, Ain Shams University (FMASU MD120/2021). The extracted patient information was de-identified to ensure anonymity and the study adhered to the tenets of the Declaration of Helsinki.

### Data extraction and inclusion criteria

Charts of patients with a confirmed diagnosis of choroidal melanoma or choroidal metastasis during the four-year interval of 2018 to 2022 were retrospectively reviewed for the availability of OCT imaging at the time of diagnosis. The diagnosis of choroidal melanoma was based on the modified criteria proposed by Shields et al. [16] in addition to documented growth on serial ultrasonography and/or fundus photography, while the diagnosis of choroidal metastasis was based on the clinical history, documentation of primary malignancy elsewhere, and multimodal imaging of the choroidal tumor. We only included eyes with adequate quality spectral-domain OCT B scans (signal strength ≥ 40) within the macula (centered around the fovea) and passing through the tumor region. The availability of fundus photography was another requirement. Eyes that had concurrent macular pathology that may result in intraretinal fluid (IRF) or subretinal fluid (SRF) accumulation (e.g., diabetic maculopathy, retinal vascular occlusive disease, and macular neovascularization) and those with ocular media opacity that prevented adequate identification of individual retinal layers on OCT were excluded from the analysis.

Extracted patient data included age, sex, laterality, choroidal melanoma tumor dimensions on presentation measured by B-scan ultrasonography, and primary tumor origin for choroidal metastases. Ocular clinical course, treatment modality, and follow up data were also retrieved when available. Best corrected visual acuity (BCVA) was recorded in Snellen format and later converted to the logarithm of the minimal angle of resolution (logMAR) with counting fingers, hand motion, light perception, and no light perception being designated as 2.10, 2.40, 2.70, and 3.00 logMAR, respectively. Intraocular pressure (IOP) measurements using an air puff tonometer on presentation were also recorded.

### Image grading

The qualitative analysis of OCT scans was done by two independent retina specialists with open adjudication on any disagreements until full consensus was reached. Radial, raster or line scans passing through the fovea and through the tumor region were assessed for the presence of intraretinal fluid (IRF), subretinal fluid (SRF), and BALAD. The diagnosis of BALAD was based on the previously reported [1, 3] anatomical description of an abnormal dome-shaped elevation located within the outer retina, having an outer boundary corresponding to the ellipsoid zone (EZ) and an elevated inner boundary corresponding to the ELM. In cases with indistinct EZ along the suspected lesion, the margins (corners) of the elevation were evaluated and BALAD was diagnosed if a split between the EZ and the ELM could be detected. The reflectivity of the BALAD was compared to that of the vitreous cavity and adjacent SRF compartments. Subretinal hyperreflective material (SHRM) at the floor of the BALAD and discernable suspended hyperreflective particles within its lumen were documented. The number and size of the BALAD were also assessed. The size of the BALAD was considered small if its longest horizontal diameter was smaller than that of the optic disc, otherwise, it was considered of large size.

### Statistical analysis

All data were anonymized and entries were made into an Excel (Microsoft 365) sheet. Data were analyzed using the Statistical Package for the Social Sciences version 25 (IBM, NY, USA). We presented numeric data as both mean ± standard deviation (SD) and median, and categorical variables as frequency (%). Where appropriate, the Student’s t-test was used to compare means between groups.

## Results

### Patient demographics

Our search initially identified 29 eyes, 17 with choroidal melanoma and 12 with choroidal metastasis. Of those, 10 eyes with choroidal melanoma and 1 eye with choroidal metastasis were excluded due to unavailability or poor-quality of OCT scans. A summary of the demographics and basic clinical information of the analyzed sample (17 patients, 18 eyes) are presented in Table 1. Two of the eyes with choroidal metastasis belonged to the same patient. The mean (± SD) age of the patients in the melanoma group was 57.1 (± 14) years, and in the metastasis group was 62.2 (± 10.4) years. Eyes with BALAD in both cohorts belonged to younger patients on average (melanoma: 38 years; metastasis: 58.3 ± 11 years) compared to eyes without BALAD (melanoma: 60.3 ± 12.2 years; metastasis: 66.8 ± 8.5 years, p = 0.193). For eyes with choroidal metastasis, BCVA was better on average in eyes with BALAD (1.4 ± 0.9 logMAR) compared to those without BALAD (2.0 ± 0.7 logMAR, p = 0.256). The most common primary sites of origin were the breast (7 eyes, 63.6%) and lung (2 eyes, 18.2%).

**Table 1:** Distribution of the demographics and clinical characteristics of the eyes without BALAD (17 patients, 18 eyes) and in eyes with BALAD (6 patients, 7 eyes)

	Choroidal Melanoma without BALAD	Choroidal Melanoma with BALAD	Choroidal Metastasis without BALAD	Choroidal Metastasis with BALAD
Total eyes, n	6	1	5	6
Age (years)				
• Mean ± SD	60.3 **±** 12.2	38	66.8 **±** 8.5	58.3 **±** 11.0
• Median	62.5		66	62.5
Gender, n (%)				
• Male	4 (66.7)	1 (100)	1 (20)	1 (16.7)
• Female	2 (33.3)	0	4 (80)	5 (83.3)
Laterality, n (%)				
• Right Eye	2 (33.3)	0	3 (60)	1 (16.7)
• Left Eye	4 (66.7)	1 (100)	2 (40)	5 (83.3)
Primary Origin, n (%)				
• Breast			2 (40)	5 (83.3)
• Lung			2 (40)	0
• Thyroid			1 (9.1)	0
• Carcinoma of Unknown Primary			0	1 (16.7)
Tumor Height (mm)				
• Mean ± SD	6.3 **±** 3.4	4.9		
• Median	6.1			
Tumor Basal Diameter (mm)				
• Mean ± SD	10.8 **±** 4.3	11		
• Median	9.3			
Tumor Macular Involvement, n (%)				
• Yes	5 (83.3)	1 (100)	5 (100)	6 (100)
• No	1 (16.7)	0	0	0
BCVA (logMAR) at presentation				
• Mean ± SD	1.0 **±** 1.0	1.3	2.0 **±** 0.7	1.4 **±** 0.9
• Median	0.6		2.4	1.6
IOP (mmHg)				
• Mean ± SD	13.2 **±** 2.7	14	15 **±** 0	12.2 **±** 1.5
• Median	13		15	12

BALAD: bacillary layer detachment, SD: standard deviation, mm: millimeters, BCVA: best corrected visual acuity, LogMAR: logarithm of the minimal angle of resolution, IOP: intraocular pressure

### Choroidal melanoma with BALAD

**Fig. 1:**
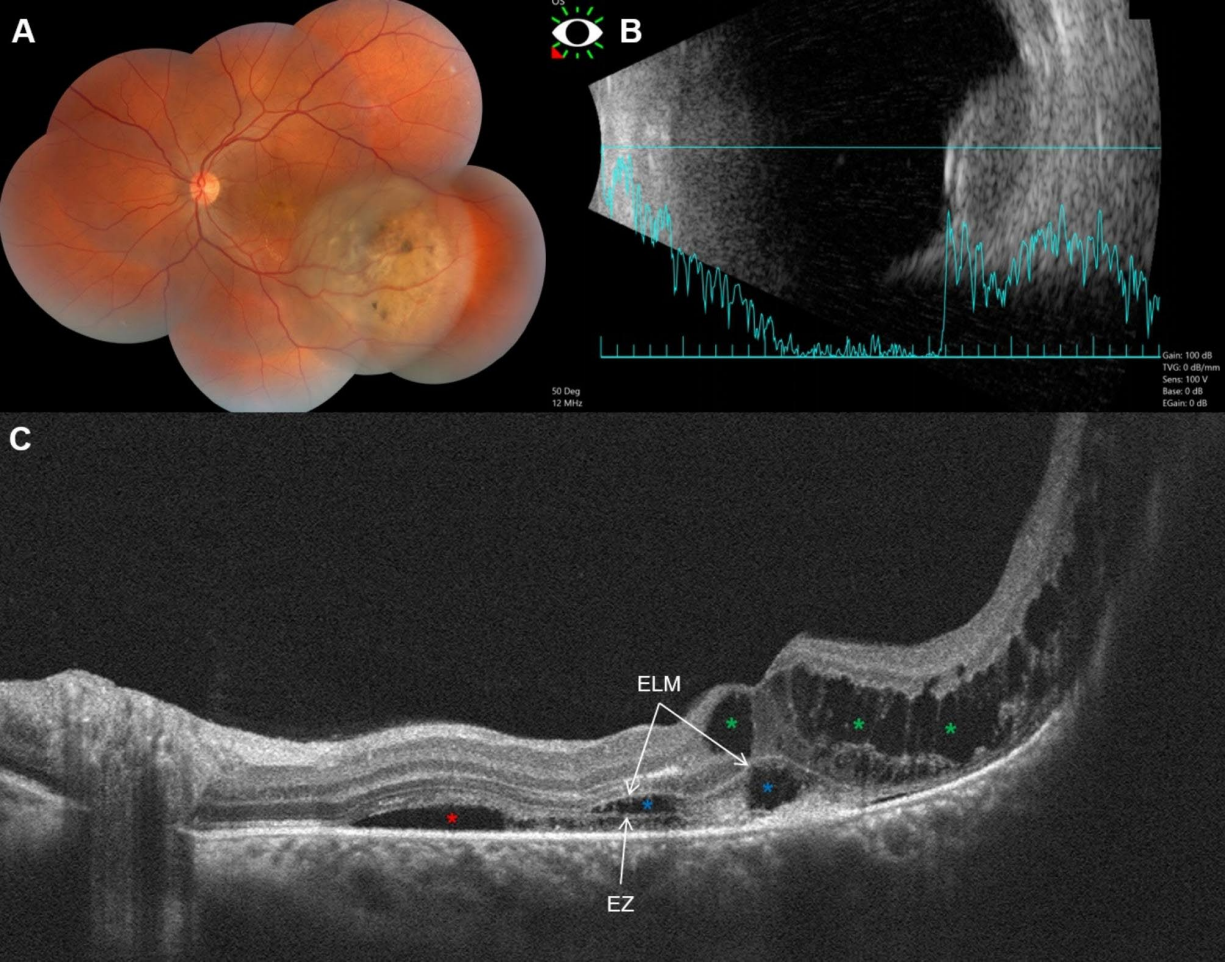
Choroidal melanoma with BALAD. Montaged fundus photography of a left eye in panel (A) depicts a lower temporal macular amelanotic mass with overlying pigment deposition and cystic changes. On B-scan ultrasonography the tumor is dome shaped with low to moderate internal reflectivity (B). Optical coherence tomography line scan passing through the fovea and the nasal edge of the tumor (C) shows localized SRF (red asterisk), diffuse cystoid edema over sloping edge of the tumor (green asterisks), and BALAD (blue asterisks). The BALAD is noted in the foveal region, adjacent to the tumor margin, and co-localizing with other fluid compartments. Hyperreflective material can be seen at the floor of the BALAD obscuring part of the EZ and RPE underneath it

All 7 eyes with choroidal melanoma had SRF, and 2 eyes (28.6%) had IRF. We could detect BALAD in 1 eye (14.3%) that belonged to a 38-year-old male patient (Fig. 1). On examination, he had a BCVA of 20/20 in the right eye and 20/400 in the left eye. The fundus of the left eye had an amelanotic dome-shaped mass within the temporal macula (Fig. 1A), the height of the which was 4.9 mm, and the basal diameter was 11 mm on B-scan ultrasonography (Fig. 1B). Optical coherence tomography revealed localized SRF and cystoid IRF collections overlying the tumor, together with evidence of foveal BALAD adjacent to the tumor margin (Fig. 1C). Discernable suspended hyperreflective particles and SHRM were detected in the lumen and the floor of the BALAD, respectively. The patient elected to undergo plaque brachytherapy in his original care facility and no follow-up data was available.

### Choroidal metastasis with BALAD

All eyes with choroidal metastasis had SRF and 4/11 eyes (36.4%) had IRF overlying the tumor. Bacillary layer detachment could be detected in 6/11 eyes (54.5%). Eyes with BALAD had metastases that originated from primary breast malignancy in most cases (5/6, 83.3%), while 1 eye belonged to a patient that had a carcinoma of unknown primary. In all eyes with BALAD, the tumor involved the macular region (Table 1).

**Fig. 2:**
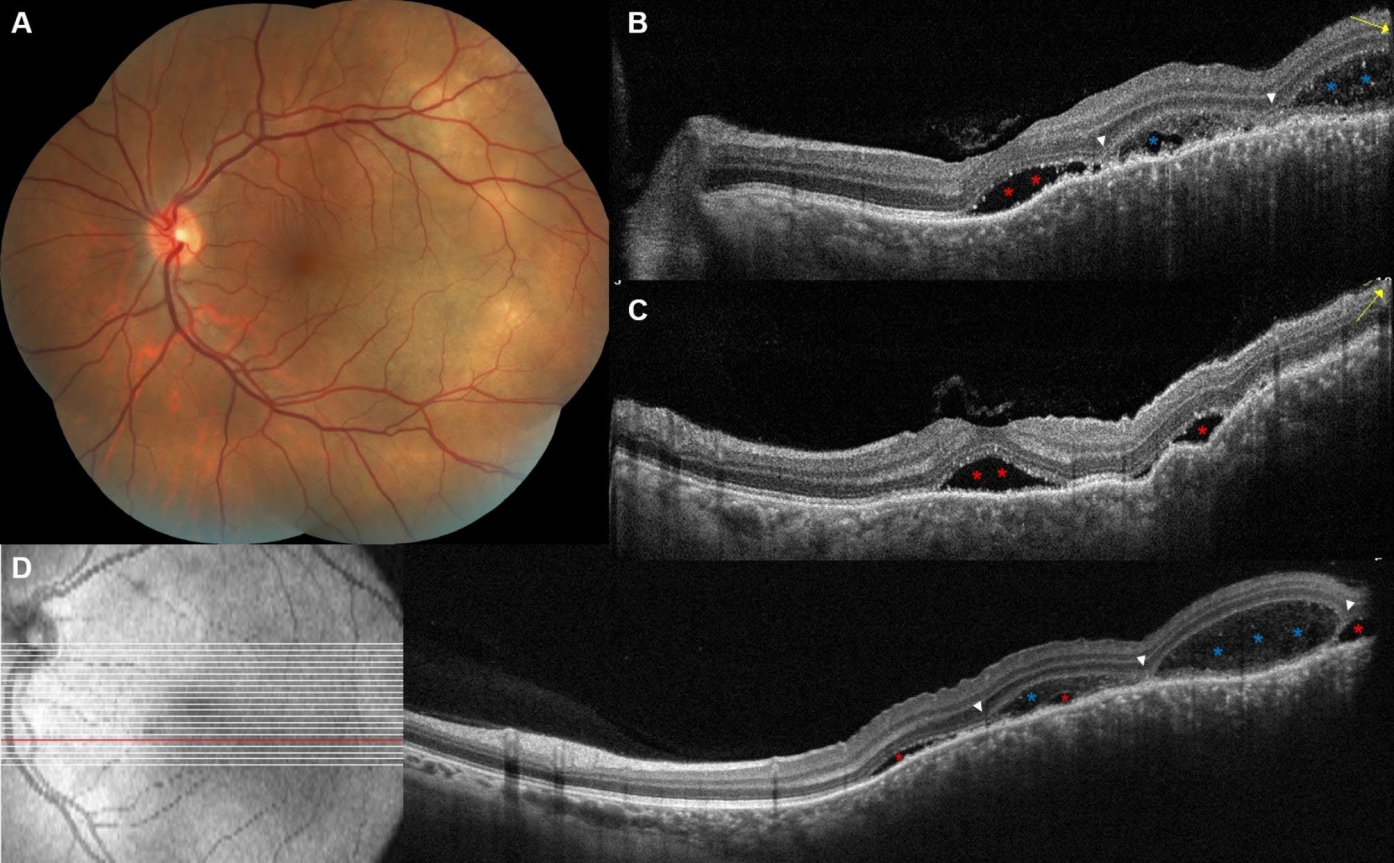
A case of choroidal metastasis with BALAD in a 40-year-old female patient with breast cancer. Fundus examination revealed multiple temporal choroidal masses with overlying SRF involving the foveal region (A). Radial scans centered over the fovea (B, C) and a raster scan over the lower macula (D) show SRF accumulation (red asterisks) involving the fovea and temporal macula, and BALAD (blue asterisks) localized to the lower parafoveal and lower temporal macular areas. White arrowheads point towards the site of splitting between the EZ and the ELM. Compared to SRF which appear as clear hyporeflective spaces, BALAD consistently demonstrates moderately hyperreflective material (turbidity) within the lumen. Yellow arrows point to the direction of the radial OCT B scan

**Fig. 3:**
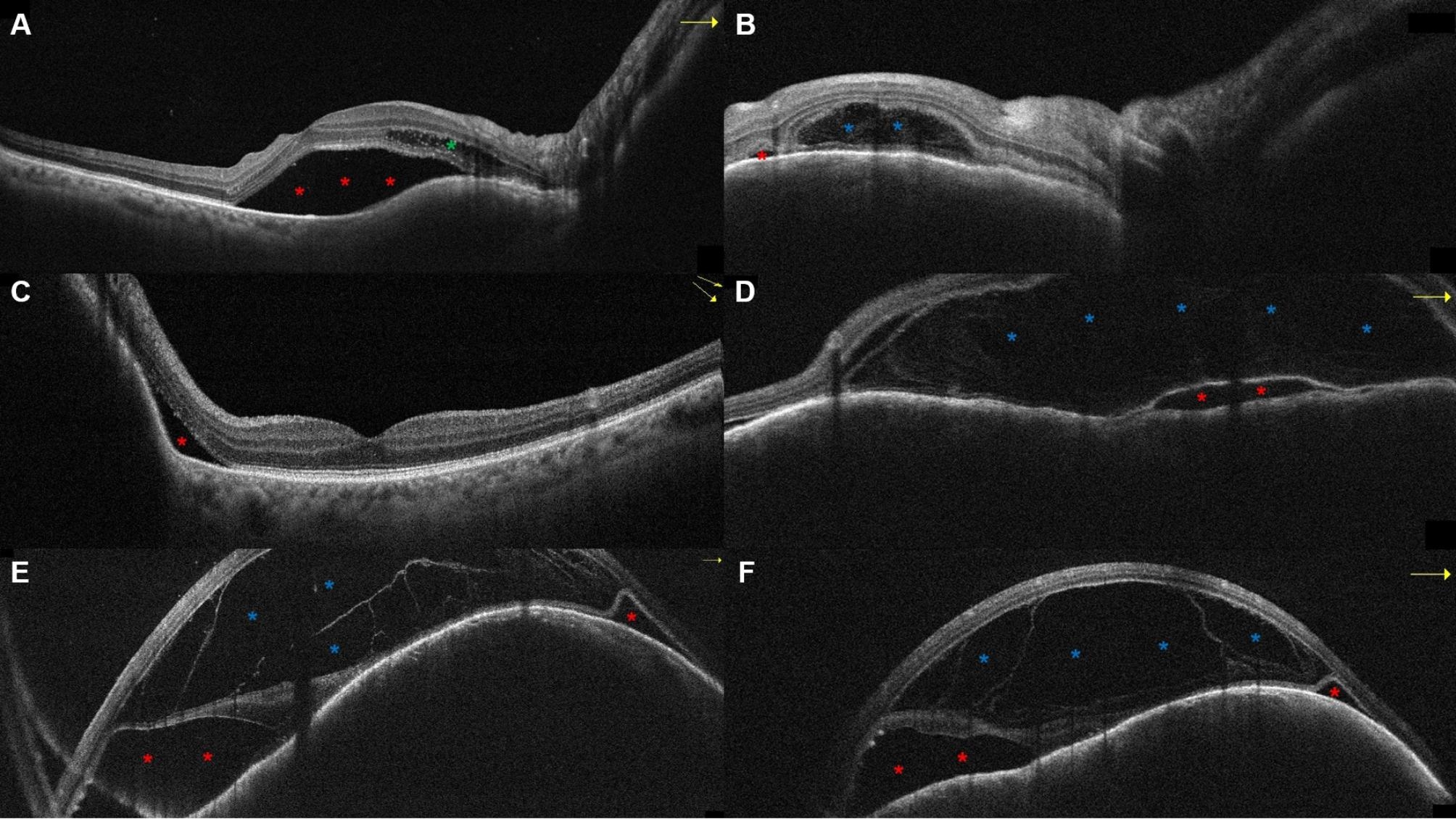
Representative cases of OCT appearance of BALAD in eyes with choroidal metastasis. A 67-year-old female patient with multiple choroidal metastases in both eyes from breast cancer. The right eye scans (A,B) show SRF beneath the fovea and nasally (red asterisks), parafoveal IRF (green asterisk), and a single small BALAD in the nasal macula within the peripapillary region (blue asterisk). The left eye scans (C,D) show nasal SRF (red asterisk) and a single large BALAD in the upper peripapillary region (blue asterisk). A 51-year-old with choroidal metastasis in the left eye from breast cancer, with OCT scans (E,F) showing extensive SRF accumulation (red asterisk) and a single large BALAD in the upper perifoveal region (blue asterisk). Multiple vertical hyperreflective lines are seen traversing the thickness of the BALAD. Yellow arrows point to the direction of the OCT B scan

The BALAD was located underneath the fovea (2 eyes, 33.3%), in the perifoveal area (2 eyes), or in the peripapillary region (2 eyes). It co-localized with the SRF in all eyes and did not localize with the IRF in 2/2 eyes. The reflectivity within the BALAD was consistently higher than that of the vitreous and adjacent SRF compartments in all eyes, and discernable suspended hyperreflective particles could be detected within the BALAD in 4/6 eyes (66.6%). We could not detect SHRM at the floor of the BALAD in any of the cases. Regarding the morphology of BALAD, it was single and large in 4/6 eyes (66.6%), multiple and small in 1 eye (16.7%), and single and small in 1 eye. The inner boundary formed by the ELM and the outer boundary formed by the EZ were discernable along the course of BALAD in all eyes (Figs. 2 and 3). One eye (Fig. 3E, F) belonging to a 51-year-old female had multiple vertical hyperreflective lines traversing the thickness of the BALAD from the EZ to the ELM, dividing the BALAD into separate compartments.

Two patients (3 eyes, 50%) were treated and followed up at the same study location, with a duration of follow up of 13 and 18 months. Treatment was by external beam radiotherapy in both patients. The three eyes showed improvement of BCVA from logMAR 2.4, 0.8 and 0.2 to logMAR 0.5, 0.5 and 0, respectively. Follow-up OCT scans were unavailable or inadequately tracked to original scans to allow comparison in two eyes; scans including the area with BALAD (foveal) were only available for 1 eye and showed complete resolution of the BALAD with partial resolution of the adjacent SRF 2 months after therapy.

## Discussion

Our current understanding of BALAD is in its early stages, with multiple questions that remain unanswered regarding its etiology, pathology, incidence with different ocular diseases, and prognostic implications [1]. In this imaging analysis of 18 eyes with malignant choroidal tumors, we demonstrate that BALAD is not that uncommon in eyes with choroidal malignancy, occurring in over half of the studied eyes with choroidal metastasis and in 1 of 7 eyes with choroidal melanoma. We also analyzed the patient demographics, tumor characteristics and OCT features of BALAD among our studied cohort, the latter supplements our recognition of this new entity across different pathologies.

Studies on BALAD detection have majorly focused on its prognostic implications in macular neovascularization (reported incidence range, 4.5 − 7.4%) [2–5, 17] and inflammatory choroidal pathologies such as Vogt-Koyanagi-Harada disease (reported incidence, 94.9%) [6, 18]. Case reports or small case series have also described BALAD with other inflammatory ocular pathologies as acute posterior multifocal placoid pigment epitheliopathy [13, 19, 20] and serpiginous-like choroidopathy associated with tuberculosis [12, 21], infiltrative ones such as unilateral diffuse uveal melanocytic proliferation [15] and choroidal metastasis [14], degenerative lesions as macular telangiectasia that was associated with acute leakage and hemorrhage [22], and blunt eye trauma (reported incidence, 8% [7]). The incidence of BALAD with most of these pathologies is less recognized owing to the lack of structured analyses. Here, we provide preliminary incidence data on BALAD occurrence with the two most common types of choroidal malignancy and demonstrate an incidence of 54.5% and 14.3% with choroidal metastasis and melanoma, respectively. Recently, Guner et al. [23] reported on the SRF characteristics of a large sample of choroidal melanomas (236 patients). They found the incidence of BALAD to be 28.4%, and that it was more common in medium-sized tumors with larger SRF, which is consistent with our described case of medium-sized malignant melanoma and BALAD.

Agarwal [24] postulated that retinal acute fluid accumulation is responsible for the photoreceptor fracture seen with BALAD, and that the rapidity by which fluid collects in the outer retina is the most important determinant of BALAD occurrence. However, this does not explain the development of BALAD in eyes with gradual fluid accumulation, as would presumably occur with choroidal malignancy such as the cases described here. Ramtohul et al. [1] theorized that the pathophysiology of BALAD is related to breakdown of the RPE component of the outer blood-retinal barrier, which may be related to choroidal ischemia and compression of the choriocapillaris. Previous reports utilizing enhanced depth imaging OCT to study eyes with choroidal melanoma [25] and choroidal metastasis [26] have documented shared features between both tumor types, including choriocapillaris thinning/compression, RPE attenuation/atrophy, and structural loss of the photoreceptors’ interdigitation zone and EZ. Nevertheless, without sufficient histopathological evidence, the inciting feature for BALAD and the shared pathophysiology between both tumor types remains to be confirmed.

The appearance of BALAD in our cohort was consistent with the description found in previous reports [1, 6, 27]. The lesion could be described as a dome-shaped space that develops within the outer retina between two hyperreflective bands, the EZ externally and the ELM internally. Increased reflectivity within the BALAD compared to the vitreous and adjacent SRF was a consistent finding in all our cases. In the literature review by Ramtohul et al. [1], increased reflectivity within the BALAD was found in 90.8% of the reported cases. In the same review, suspended hyperreflective granules were noted in 77.1% of the cases; similarly, we could detect these particles in 5 of the 7 eyes with BALAD (71.4%). The BALAD co-localized with SRF in all 7 eyes and with IRF in 1/3 eyes (33.3%) across our sample. This is consistent with the findings by Ramtohul et al. [1] where BALAD was more commonly detected adjacent to SRF (77.5%) than IRF (5.6%). SHRM was only noted in our described case of BALAD with choroidal melanoma, which appeared to be continuous with hyperreflective material within the BALAD (Fig. 1C). In the recent report but Guner et al. [23] 58% of the eyes with BALAD were associated with heterogenous hyperreflective material. The size and number of BALAD varied among our cases, some were subtle and small (Figs. 1C and 2D), and others were very large (Fig. 3D, E), with the single large BALAD being the most detected morphology. Of note, one eye with choroidal metastasis and a single large overlying BALAD exhibited a distinct appearance of multiple vertical hyperreflective lines or septa, dividing the BALAD into separate compartments (Fig. 3E, F). These septa were long recognized in the OCT scans of eyes with Vogt-Koyanagi-Harada disease [28], and were recently described as part of the BALAD presentation. Hypotheses regarding their composition include fibrin-like material or migrating mitochondria [1, 13].

Our study provides preliminary information on the incidence and imaging features of BALAD with two different ocular malignancies. The study of BALAD in malignant choroidal tumors provides a unique opportunity for its longitudinal analysis with the slowly evolving fluid accumulation and gradual resolution after therapy. Furthermore, enucleated eyes for large choroidal tumors that had BALAD could serve in the histopathological analysis of these lesions. There are, however, limitations to our study. This was a retrospective study, which is a common theme to all currently available studies on BALAD owing to its recent description. Considering the dynamic evolution of BALAD, the retrospective review of records would be limited in picking up all cases and determining true incidence, as well as in following up the lesions and determining their prognostic implications. Further, the small sample size limits the accurate derivation of incidence rates and underpowers the analysis in detecting small differences between eyes that developed BALAD and those that did not in order to suggest any prognostic implications of BALAD. Future prospective studies or post-hoc analyses of existing datasets with larger samples are warranted to address these issues.

In conclusion, we demonstrate the occurrence of BALAD with the two most common types of intraocular malignancy. We report an incidence rate of 54.5% in eyes with choroidal metastasis and 14.3% in eyes with choroidal melanoma. The reported OCT features of BALAD in the setting of choroidal malignancy supports our recognition of this new feature across different ocular pathology.

## Data Availability

Derived data supporting the findings of this study are available from the corresponding author on request.

## Abbreviations

BALAD: bacillary layer detachment
BCVA: best corrected visual acuity
ELM: external limiting membrane
EZ: ellipsoid zone
IOP: intraocular pressure
IRF: intraretinal fluid
LogMAR: logarithm of the minimal angle of resolution
mm: millimeters
OCT: optical coherence tomography
RPE: retinal pigment epithelium
SD: standard deviation
SRF: subretinal fluid
